# A Story of Fifty Years Achievement

**Published:** 1916-10

**Authors:** 


### A Story of Fifty Years Achievement.

Fifty years ago the house of Parke, Davis & Co. was born in a drug store. This year the managers are celebrating the golden jubilee, and in honor of the occasion they have issued a handsome booklet which gives a brief history of the early struggles and later attainments of this well known drug house. The book is interesting from the first page to the last, and it is also expressive of the character of the business. It contains a well written, modest, straightforward statement of difficulties encountered and overcome which should inspire courage in the heart of any struggling man or firm. The book is profusely illustrated with interesting pictures of the leaders of the past, directors and officers of today; the laboratory in Hounslow, England, and the Canadian Laboratory in Walkerville, Ontario; the headquarters' executive staff; buildings and scenes of Parkdale Farm, where the animals are kept for the production of antitoxic serums; the research laboratory; the American branch houses and their managers, some of the foreign managers and the foreign branches.

By contrast the most interesting pictures are those of the house where the business was born in 1866 and that occupied by it today. It is rather hard to believe that from such a tiny accrn so large an oak has grown, but it is true and today its branches (including a dozen or so in the United States) spread to nearly every part of the civilized globe.

Upon a careful study of the faces of the leaders in the past and of the present directors, officers and executive staff, one comes to understand this phenomenal growth. The firm of Parke, Davis & Co. has prospered because it has strong, forceful, capable men behind it; men who expressed their own characters through the quality of the products they manufactured, and who by their honest dealings with their patrons have inspired not only the respect but the love and confidence of those they serve. Today they have no superiors and few equals. They are worthy of a whole dictionary of encomiums, for they have been real world benefactors. May they continue to grow in the next half century even beyond their fondest expectation, and may other business concerns of the country profit by their example of industry, perseverance, integrity and the application of the Golden Rule.

